# Cyclin B2 overexpression promotes tumour growth by regulating jagged 1 in hepatocellular carcinoma

**DOI:** 10.18632/aging.203979

**Published:** 2022-03-29

**Authors:** Yening Xiao, Jiamei Ma, Chunliu Guo, Danni Liu, Jing Pan, Xiaoxi Huang

**Affiliations:** 1 Department of Gastroenterology, Central South University Xiangya School of Medicine Affiliated Haikou Hospital, Haikou 570028, China

**Keywords:** hepatocellular carcinoma, CCNB2, JAG1, proliferation, migration

## Abstract

Background: Our previous study showed that Cyclin B2 (*CCNB2*) is closely related to the occurrence and progression of hepatocellular carcinoma (HCC).

Aim of the study: This study aimed to clarify the effect of *CCNB2* gene silencing on tumorigenesis in nude mice and to detect the potential mechanism.

Methods: The effect of *CCNB2* on HCC was tested *in vivo*. The downstream target genes of CCNB2 were predicted by proteomics and confirmed by western blot assay. The regulatory functions of *CCNB2* in the proliferation and migration of HCC cells were determined through functional recovery experiments. The expression of the downstream target genes of *CCNB2* was detected by immunohistochemistry.

Results: Knockdown of *CCNB2* decreased tumour formation rate and tumour volume and weight and inhibited tumour proliferation. A total of 130 differentially expressed proteins were detected by proteomics, and Jagged 1 (JAG1) was predicted as the potential downstream target of CCNB2. Western blot assay revealed that CCNB2 and JAG1 expression was significantly correlated in HCC cells. The results of functional recovery experiments suggested that *CCNB2* knockdown weakened the proliferation and migration ability of HCC cells, while *JAG1* overexpression restored this ability of HCC cells that was weakened by *CCNB2* knockdown. Immunohistochemistry showed that JAG1 expression was higher in HCC tissues than in paracancerous tissues and was related to tumour size and number and tumour thrombus formation.

Conclusions: The proliferation of HCC cells *in vivo* was inhibited by *CCNB2* knockdown. *CCNB2* may accelerate the proliferation and metastasis of HCC cells by increasing *JAG1* expression.

## INTRODUCTION

Hepatocellular carcinoma (HCC), a widely occurring malignancy, is the most common histological form of primary hepatic carcinoma. According to the global data in 2018, HCC ranked sixth among malignancies and fourth among mortality associated with malignancies [1]. In recent years, with the continuous advancement in the medical field, significant progress has been made in the diagnosis and treatment of HCC. However, despite this progress, the 5-year survival rate of patients with HCC is still not ideal even after treatment with existing advanced technologies [2, 3]. It is therefore necessary to further investigate the pathogenesis of HCC, find better diagnostic markers and drug targets and improve the level of diagnosis and treatment of HCC.

The cell cycle is the basic process of cell biological activities. Abnormal cell cycle regulation, especially the cyclin family proteins (cyclins), is correlated with the occurrence and progression of various types of malignancies. Cyclin B2 (CCNB2) is a member of the cyclin family and binds to CDK2, which enables cells to progress toward the G2/M phase [4]. Abnormal *CCNB2* expression may cause malfunctions of the G2/M checkpoint, leading to gene mutation, structural changes in the chromosome and abnormal growth of cell number, thus leading to the development of tumours [5]. *CCNB2* is significantly overexpressed in colon carcinoma [6], gastric carcinoma [7] and other cancer tissues, and this overexpression is closely correlated with a variety of malignant biological functions.

In our preliminary research, we found that the up-regulation of *CCNB2* was related to the prognosis of HCC through the gene expression profile interaction analysis (GEPIA) database [8]. *CCNB2* may accelerate the proliferation and migration of human hepatoma cells and inhibit their apoptosis.

Based on previous research and results of tumour formation experiments in nude mice, the present study focused on CCNB2 as the primary research objective. Proteomic analysis showed that the Notch signalling pathway ligand molecule Jagged 1 (JAG1) might be a potential target of CCNB2, and the regulatory effect of CCNB2 on JAG1 was confirmed.

## MATERIALS AND METHODS

### Clinical specimens and cell lines

From January 2018 to September 2020, tissues were collected from 82 patients with HCC and 20 patients with corresponding paracancerous lesions at Haikou Hospital Affiliated to Xiangya Medical College of Central South University. All patients were diagnosed to have HCC by pathological examination. The clinical and pathological data of 82 patients with HCC were collected. All patients had not received preoperative treatment such as hepatic arterial chemoembolization, local radiofrequency ablation, targeted therapy, chemotherapy or radiotherapy and were pathologically confirmed to have HCC after surgery. Patients with HCC together with other neoplasms or with positive tumour margins were excluded. The study was approved by the ethics committee of Haikou Hospital Affiliated to Xiangya Medical College of Central South University.

The human HCC cell line (Huh-7) used in this study was obtained from the Cell Bank of the Chinese Academy of Sciences in Shanghai, China. Huh-7 cells were cultured in Dulbecco’s Modified Eagle’s Medium (Gibco, Carlsbad, CA, USA).

### Cell transfection

*CCNB2* small interfering ribonucleic acid (siRNA) (RNAi:5ʹ-CAAGAATGTGGTGAAAGTA-3ʹ) and negative lentivirus were obtained from Shanghai GeneChem Co., Ltd., (China). The experimental groups were as follows: KD group: Huh-7 cells infected with *CCNB2*-siRNA lentivirus; NC group: Huh-7 cells infected with CON077 negative control virus; and CON group: Uninfected empty Huh-7 cells.

The expression of the *CCNB2* gene was determined by fluorescence microscopy approximately 72 h after infection. The fluorescence rate indicated the positive infection rate. The cells were stable, and the infection efficiency reached that of the standard group, which could be used for later experimental research.

### Quantitative real-time PCR (qPCR)

TRIzol reagent (Shanghai Pufei Biotech Co., Ltd., China) was used to extract total R-NA, and the Bulge-Loop™ miRNA qPCR Primer Set (Guangzhou Ruibo Biotechnology Co., Ltd., China) was used for reverse transcription in accordance with the manufacturer’s protocol. qRT-PCR was performed on a real-time PCR instrument (Roche, Switzerland) with SYBR Master Mix (TaKaRa, Dalian, China). *ACTB* was used as an internal control. The primer information is as follows: *CCNB2*-forward primer: 5ʹ-CAACCCACCAAAACAACA-3ʹ; *CCNB2*-reverse primer: 5ʹ-AGAGCAAGGCATCAGAAA-3ʹ; *ACTB*-forward primer: 5ʹ-GCGTGACATTAAGGAGAAGC-3ʹ; *ACTB*-reverse primer: 5ʹ-CCACGTCACACTTCATGATGG-3ʹ.

### Nude mice tumorigenesis experiment

The animal experiment was approved by the ethics committee of Haikou Hospital Affiliated to Xiangya Medical College of Central South University. All animal experiments were conducted in accordance to ARRIVE guidelines for animal experiments. Twenty nude mice were randomly distributed into two groups. The experimental groups were as follows: (1) NC group (control group): mice (numbers 1–10) were injected subcutaneously with Huh-7 cells without *CCNB2* knockdown and (2) KD group (experimental group): mice (numbers 11–20) were injected subcutaneously with Huh-7 cells with *CCNB2* knockdown. Tumour development was observed over the next 4 weeks, and tumour size and the weight of mice were measured every 5 days. Tumour volume was calculated using the formula: 0.5 × (length × width^2^) [9]. Based on actual tumour formation and the guidelines of the animal ethics committee, the tumour-bearing nude mice were euthanised, and the tumours were entirely removed and weighed.

### Tandem mass tag marker quantification

SDT lysate (Shanghai GeneChem Co., Ltd.) was added to the sample for protein extraction. Next, FASP enzymatic hydrolysis was performed for the extracted protein after SDS-PAGE. Tandem mass tag (TMT) labelling was performed according to the instructions provided in the TMT labelling kit (Thermo Fisher Scientific, USA). The labelled peptides of each group were mixed, and high-pH reversed-phase fractionation was performed using an HPLC system (Thermo Fisher Scientific). The samples were eluted with the nanoscale flow rate EASY-nLC system (Thermo Fisher Scientific), and the chromatographically separated samples were analysed with a Q-Exactive Plus mass spectrometer (Thermo Fisher Scientific). Mascot 2.6 (Matrix Science, UK) and Proteome Discoverer 2.2 (Thermo Fisher Scientific) were used for quantitative analysis.

### Bioinformatics analysis

Blast2GO [10] was used for Gene Ontology(GO) annotation, and KOALA (KEGG Orthology And Links Annotation) [11] software was used for Kyoto Encyclopedia of Genes and Genomes(KEGG) annotation. Subsequently, GO and KEGG enrichment analysis was performed. The software WoLF PSORT (https://wolfpsort.hgc.jp/) [12] was used for performing projection analysis of different proteins. The Interpro database was used to annotate the functional domains of differential proteins. The expression levels of the samples were graded by Matplotlib software [13], and the hierarchical heat map of clustering was established. An IPA pathway analysis (https://www.ingenuity.com) was conducted on differential proteins by using keywords such as “proliferation,” “invasion,” “apoptosis,” “cell cycle progression,” and “tumour cell immune response”.

### Parallel reaction monitoring targeted quantification

The extracted proteins were analysed by mass spectrometry. Parallel reaction monitoring (PRM) master ion screening was performed using Proteome Discoverer 2.1 software (Thermo Fisher Scientific) and Mascot 2.6 server. PRM was analysed by the EASY-nLC system (Thermo Fisher Scientific) and a Q-Exactive mass spectrometer (Thermo Fisher Scientific).

### Western blot (WB)

Total protein extraction was performed using RIPA lysate (Beyotime, Shanghai, China), and SDS-PAGE was then performed. The protein was transferred to a PVDF membrane using an electrical transfer device (Shanghai TANON Technology Co., Ltd., China). After blocking the membrane, it was incubated with primary antibody and secondary antibody successively. The following primary antibodies were used: rabbit anti-human CCNB2 antibody (Abcam, USA) and rabbit anti-human JAG1 antibody (CST, USA). X-ray imaging was performed using the CST 20X Lumiglo® Reagent and 20X spectroscopy #7003 kit (CST). The relative optical densities of all bands were quantified by GelPro Analyzer (Media Cybernetics, Silver Spring, MD, USA). All results were normalized to the internal standard (β-actin) [14].

### Cell function experiment

The experimental groups were as follows: KD+OE group: Huh-7 cells were infected by LV-*JAG1* and LV-*CCNB2*-RNAi lentiviruses; OE+NC group: Huh-7 cells were infected with LV-*JAG1* and CON077 negative control virus; KD+NC group: Huh-7 cells were infected with LV-*CCNB2*-RNAi and Con382 negative control virus; and NC+NC group: Huh-7 cells were infected with CON382 negative control virus and CON077 negative control virus.

The CCK-8 kit (Dojindo Laboratories, Japan) was used to detect cell viability. The cells were seeded onto 96-well plates (Corning, USA) at the density of 2000 cells/well. Next, 10 μL CCK-8 reagent was added before the termination of cell culture. After 4 h of incubation, the OD value was measured at 450 nm by using a microplate analyser (Thermo Fisher Scientific).

The appropriate numbers of chambers were placed in a new 24-well plate (Corning, USA). In the upper chamber, a serum-free cell suspension of 10^5^ cells/well was added, while in the lower chamber, 600 μL of 30% FBS medium (Ausbian, Australia) was added, and the plate was incubated for 24 h at 37° C. All compartments were fixed in a 4% paraformaldehyde fixation solution for 30 min. The cells were stained with crystal violet solution for 3 min and then photographed and counted using an inverted microscope (Shanghai Caikon Optical Instrument Co., Ltd., China).

### Immunohistochemistry (IHC)

The samples were dewaxed and hydrated with xylene and ethanol successively. An antigen repair solution was used to repair antigen in the Lab Vision™ PT Module. After blocking, the samples were incubated successively with primary antibody and secondary antibody. The rabbit anti-human JAG1 antibody was used as the primary antibody. After colour development with the DAB chromogenic solution, counterstaining with haematoxylin was performed, after which the sections were dehydrated with ethanol and xylene and sealed with resin.

All specimens were histologically reviewed by two independent pathologists. A positive immune reaction was indicated by brown-stained cells [14]. The percentage of positive cells was scored as follows: 0, 0% positive cells; 1, 1%–25% positive cells; 2, 26%–50% positive cells; 3, 51–75% positive cells; 4, 76%–100% positive cells. The staining intensity was scored as follows: 0, negative; 1, weak; 2, medium; and 3, strong. The final expression score was calculated by multiplying the percentage of positive cells by the fraction of staining intensity on the scale of 0 to 12. Samples with a score of > 6 were considered to have high expression, while those with a score ≤ 6 were considered to have low expression [14].

### Statistical analysis

Statistical analysis was conducted with GraphPad Prism 8.0 (GraphPad Prism, La Jolla, CA, USA) and SPSS 25.0 (SPSS, Inc. Chicago, IL, USA). Quantitative data with normal distribution were expressed as mean ± standard deviation (x±s), and comparison between two groups was performed using two independent sample t-test. Quantitative data with skewed distribution were expressed as median (first quartile, third quartile) [M (QL, QU)], and comparison between two groups was performed using the Mann-Whitney U test. The relationship between JAG1 expression and clinicopathological characteristics of patients with HCC was determined using the chi-square test. A p value of < 0.05 was considered to be statistically significant.

## RESULTS

### *CCNB2* enhances tumour growth in nude mice

**Figure 1:**
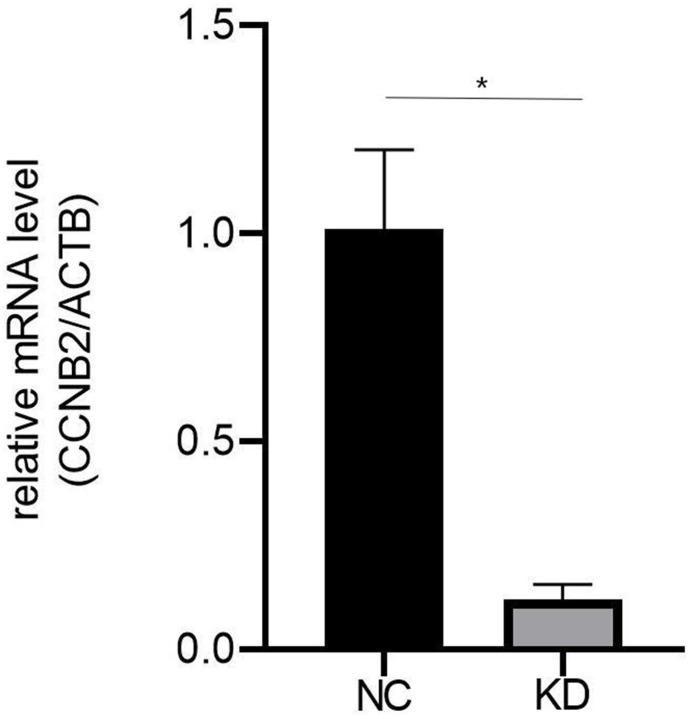
**The qRT-PCR results showed that the expression level of CCNB2 in Huh-7 cell line of CCNB2-RNAi group was significantly inhibited.** **p < 0.01.

As shown in Figure 1, the expression level of *CCNB2* was considerably reduced in Huh-7 cells transfected with *CCNB2* siRNA as determined by qPCR (KD 0.121 ± 0.036 vs. NC 1.012 ± 0.190; p < 0.01), and the interference efficiency of the KD group was 87.9%.

**Figure 2:**
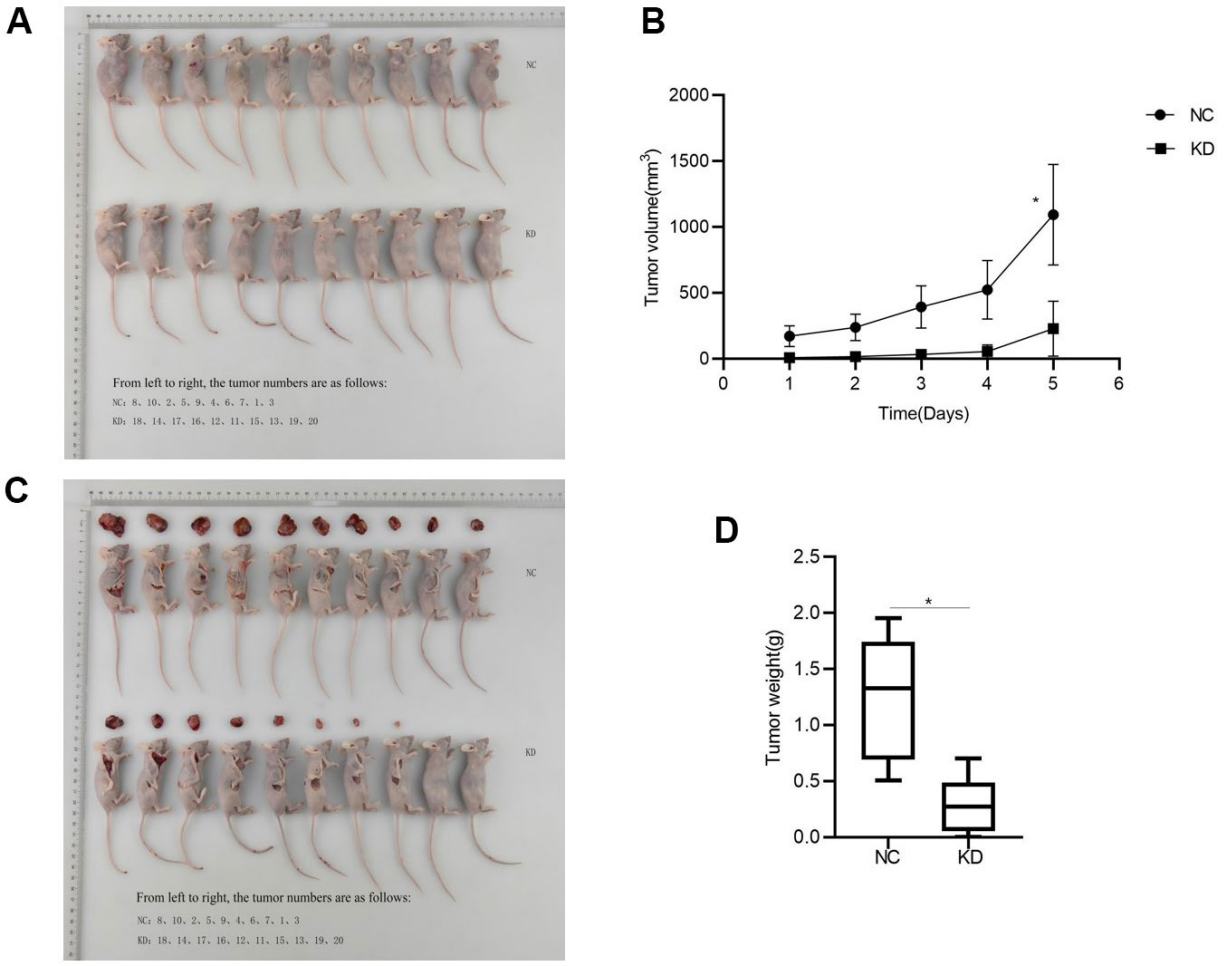
**CCNB2 enhances tumour growth in nude mice.** (**A**) Tumour formation in nude mice, the top row is NC group, the bottom row is KD group. (**B**) Changes of tumour volume in nude mice at five different time points. (**C**) Tumour of NC group and KD group at the 26th day. (**D**) Tumour weight in nude mice. *p < 0.05.

Huh-7 cells with *CCNB2* knockdown and Huh-7 cells without *CCNB2* knockdown were injected subcutaneously into the experimental group (KD group) and the control group (NC group), respectively, to observe tumour formation. After 25 days, eight nude mice in the experimental group developed tumours, and the tumour formation rate of this group was 80%. All nude mice in the control group developed tumours, and the tumour formation rate of this group was 100% (Figure 2A). These results suggest that *CCNB2* knockdown could reduce the tumour formation rate in nude mice and may inhibit the occurrence of HCC.

After the inoculation of Huh-7 cells in nude mice, the tumour volume of the NC group at five time points (6, 11, 16, 21 and 26 days) was 186.58 (99.91-240.34) [M(P25-P75)] mm^3^, 265.74 (139.55-326.00) [M(P25-P75)] mm^3^, 431.78 (216.69-528.06) [M(P25-P75)] mm^3^, 549.67 (291.7-719.26) [M(P25-P75)] mm^3^ and 1201.39 (619.57-1363.32) [M(P25-P75)]mm^3^, respectively, while the tumour volume of the KD group was 6.21 (0.00-14.45)[M(P25-P75] mm^3^, 13.47 (0.00-33.22) [M(P25-P75)] mm^3^, 28.03 (3.91-69.06) [M(P25-P75)] mm^3^, 51.5(4.98-105.61) [M(P25-P75)] mm^3^, 215.11(45.47-383.62) [M (P25-P75)] mm^3^, respectively. A significant difference in tumour volume was observed between the two groups (p < 0.05, Figure 2B). This finding suggested that the tumour volume was reduced after knockdown of *CCNB2*. The tumour growth was inhibited following *CCNB2* knockdown, which indicates that *CCNB2* may activate HCC progression.

The tumour weight of the KD group was 0.27 (0.06–0.49) [M (P25-P75)] g and that of the NC group was 1.33 (0.69-1.74)[M (P25-P75)] g (p < 0.05, Figure 2D). Tumour weight decreased after *CCNB2* was knockdown, which suggested that tumour proliferation ability decreased following *CCNB2* knockdown. Thus, *CCNB2* may be associated with promoting the development of HCC.

### Proteomic results

**Figure 3:**
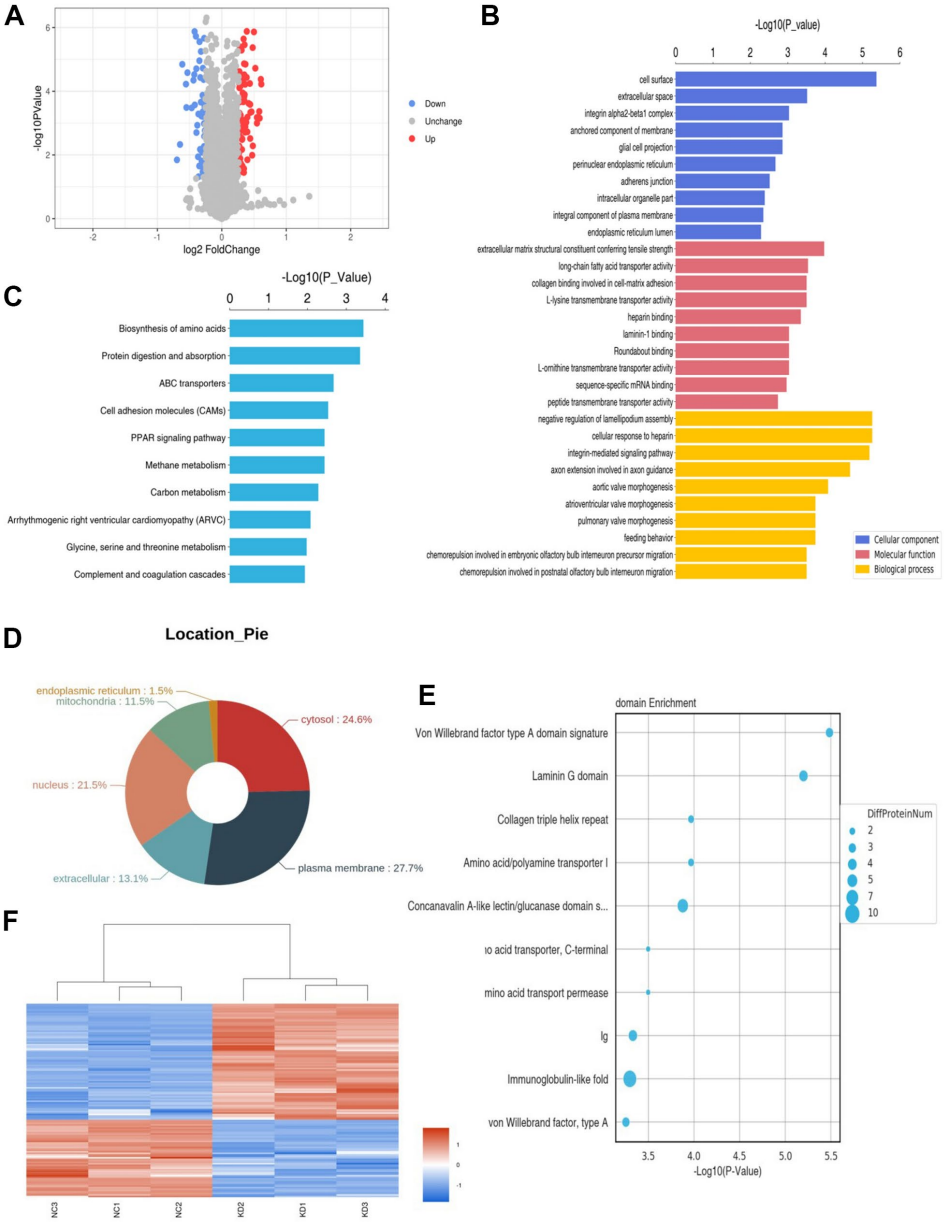
**Proteomic results.** (**A**) Differential protein volcano map. The blue circles on the left represent down-regulated proteins and the red circles on the right represent up-regulated proteins. The abscissa is the fold change; The y-coordinate is P value. (**B**) The bar chart shows the results of the top 10 GO terms with the most significant enrichment. The ordinate represents the GO function name, and the abscissa represents the P-value of enrichment significance. Blue bars represent cellular components, red bars represent molecular functions, and yellow bars represent biological processes. (**C**) The bar chart identifies the top 10 KEGG pathways with the most significant enrichment. The ordinate is KEGG pathway name and the abscissa is p-value. (**D**) Protein subcellular localization map. (**E**) The top 10 protein domains with the most significant enrichment are shown in the bubble diagram. The ordinate is the name of the structure domain, and the abscissa is the p-value. The size of the circle represents how many differential proteins are contained in the domain. (**F**) Red represents up-regulated proteins and blue represents down-regulated proteins.

A total of 7271 proteins were identified by TMT mass spectrometry. Bioinformatics analysis was used to screen proteins with expression fold change of > 1.2 (up-regulated or down-regulated) and p < 0.05 (t-test) as differentially expressed proteins. There were 130 differentially expressed proteins between the KD group and NC group, including 78 up-regulated proteins and 52 down-regulated proteins. A volcano plot was mapped to show differential proteins (Figure 3A).

GO functional annotations and enrichment analysis showed that the cell components were primarily enriched in cell surface, extracellular space, integrin alpha2-beta1 complex, anchored component of cell membrane and glial cell projection. Molecular functions were primarily involved in the structural constituent of the extracellular matrix conferring tensile strength, long-chain fatty acid transporter activity, collagen binding involved in cell-matrix adhesion, L-lysine transmembrane transporter activity and heparin binding. The biological processes were significantly enriched in negative regulation of lamellipodium assembly, cellular response to heparin, integrin-mediated signalling pathway, axon extension involved in axon guidance and aortic valve morphogenesis (Figure 3B).

KEGG pathway annotation and enrichment analysis showed that the differential proteins were primarily enriched in various metabolic pathways, especially in the biosynthesis of amino acids, cell adhesion molecules, protein digestion and absorption, PPAR signalling pathway and ABC transporters (Figure 3C).

The results of subcellular localisation analysis showed that the majority of the differential proteins were located in the plasma membrane (27.7%), followed by the cytosol (24.6%) and nucleus (21.5%), and the remaining proteins were localised in the extracellular matrix, mitochondria and endoplasmic reticulum (Figure 3D).

The results of domain annotation and enrichment analysis indicated that the differential protein domains were enriched in Von Willebrand factor type A domain signature, laminin G domain, collagen triple helix repeat, amino acid/polyamine transporter I and concanavalin A-like lectin (Figure 3E).

The cluster analysis results showed that the differential proteins could differentiate between samples with and without *CCNB2* knockdown (Figure 3F).

We selected 30 proteins from the differential proteins for PRM analysis. In the PRM analysis, proteins with the expression fold change of > 1.2 (up-regulated or down-regulated) and p < 0.01 (t-test) were considered as significant differential proteins. The KD/NC value of RAB5A was > 1, suggesting that RAB5A was up-regulated when *CCNB2* was knocked down (Table 1). The KD/NC values of PPCS and JAG1 were < 1, indicating that when *CCNB2* was knocked down, PPCS and JAG1 were down-regulated. The results of IPA for significant differential proteins showed that JAG1 was correlated with the proliferation and apoptosis of HCC cells. Based on the above results, JAG1 could probably be the downstream target of CCNB2, and it was selected for further experimental confirmation.

**Table 1:** Protein quantification results.

**Protein accessions**	**Gene name**	**KD/NC**	**P value**
P20339	RAB5A	1.44627851	0.00195452
P78504	JAG1	0.727725215	0.003030602
Q9HAB8	PPCS	0.520364985	0.006848256

### *CCNB2* knockdown inhibits *JAG1* expression

**Figure 4:**
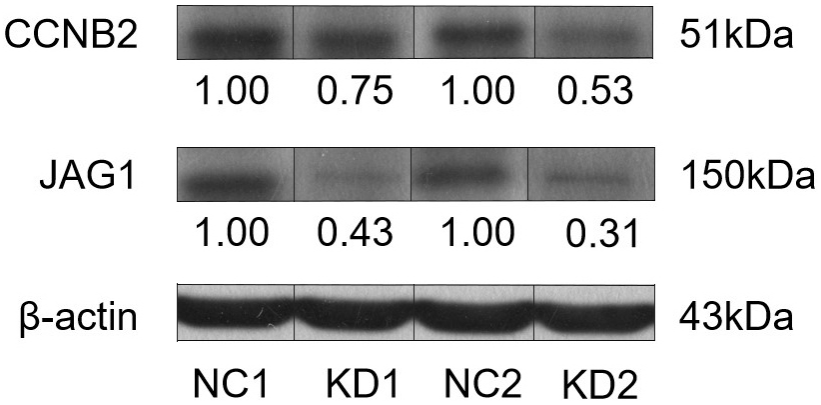
Western blot was used to detect the interaction between CCNB2 protein and JAG1 protein in the tumor tissues of nude mice.

Two nude mouse tumour tissues were randomly selected from the experimental group and control group for the WB assay. The results showed that knockdown of *CCNB2* inhibited the expression of the JAG1 protein as compared with that in the control group (Figure 4).

### CCNB2 regulates JAG1 to promote tumour cell proliferation and migration

**Figure 5:**
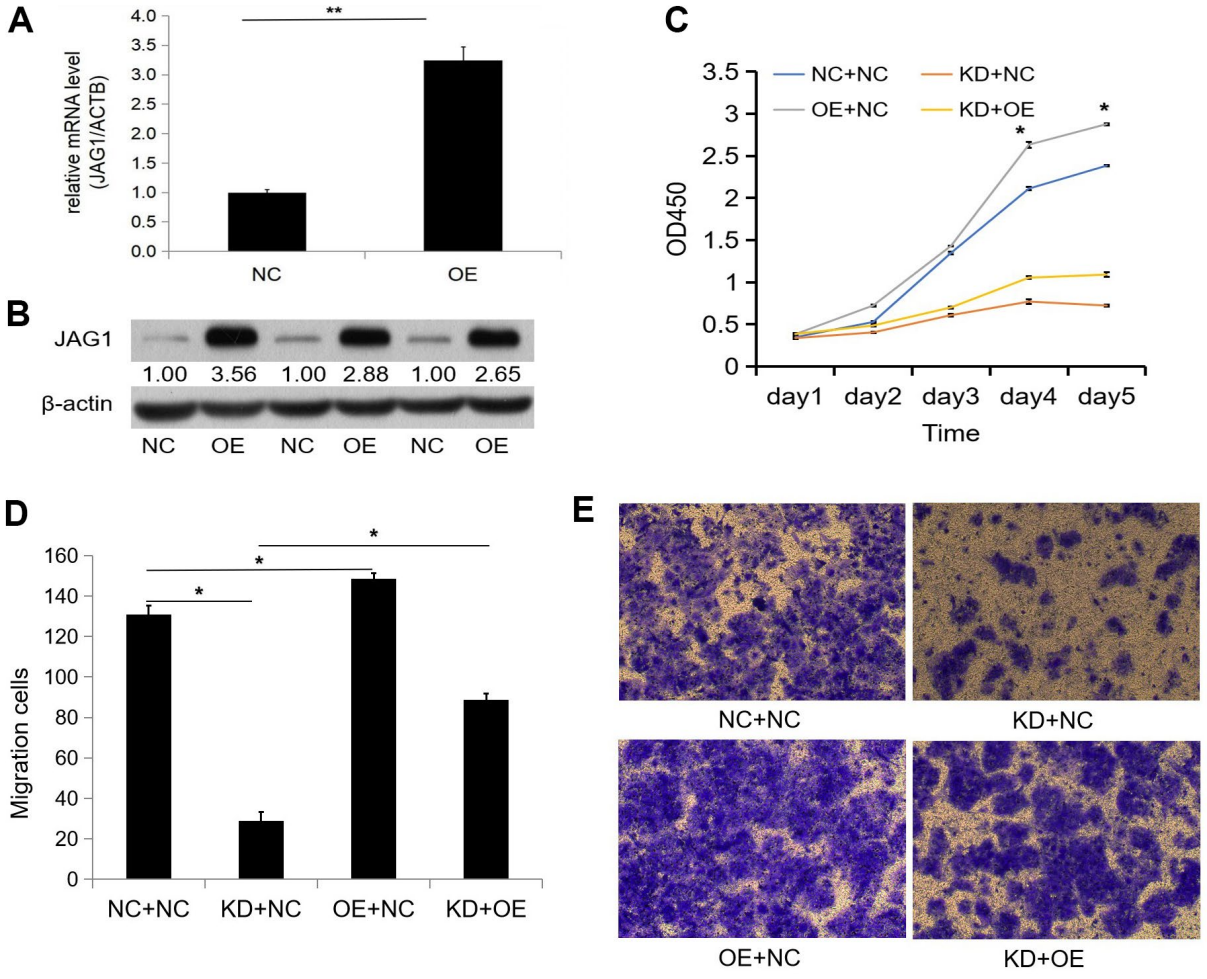
**Overexpression of JAG1 restores the inhibition of proliferation and migration caused by knockdown of CCNB2.** (**A**) The over-expression efficiency of JAG1 was detected by qPCR. (**B**) Western blot was used to observe the over-expression efficiency of JAG1 protein. (**C**) CCK-8 assays were performed after cell infection in Huh-7 cells. (**D**) Transwell assay observed the number of migratory cells in each group after Huh-7 cell infection. (**E**) Huh-7 migratory cells were detected by transwell assay. *p < 0.05, **p < 0.01.

We transfected LV-*JAG1* into the Huh-7 cell line to construct a *JAG1* functionally overexpressing cell line (OE group). The transfection efficiency was confirmed before the functional experiment. qPCR showed that the expression abundance of the *JAG1* gene in the OE group was 3.239 times higher than that in the NC group, and the expression level of the *JAG1* gene was significantly increased (p < 0.01, Figure 5A). The WB assay indicated that as compared with the NC group, the expression of JAG1 in the OE group was significantly increased at the protein level (Figure 5B). We then performed cell infection according to the experimental groups, and the experimental results showed that cell transfection was successful.

The CCK-8 assay showed that the proliferation function of Huh-7 cells in the OE+NC group (DAY4: 6.85 ± 0.09, DAY5: 7.49 ± 0.03) was enhanced as compared with that in the NC+NC group (DAY4: 6.07 ± 0.05, DAY5: 6.85 ± 0.02) on day 4 and day 5 after Huh-7 cell infection (p < 0.05), respectively. The proliferation ability of the KD+NC group (DAY4: 2.28 ± 0.09, DAY5: 2.14 ± 0.03) was weakened as compared with that of the NC+NC group (p < 0.05), while the proliferation ability of the KD+OE group (DAY4: 2.73 ± 0.04, DAY5: 2.83 ± 0.08) was partially restored as compared to that of the KD+NC group (p < 0.05, Figure 5C). The results of the CCK-8 assay indicated that *CCNB2* silencing restrained the proliferation of HCC cells, while JAG1 overexpression partially restored proliferation inhibition induced by *CCNB2* silencing. Functional recovery experiments further confirmed that *CCNB2* promoted cell proliferation by increasing *JAG1* expression.

The Transwell assay showed that the number of migratory cells in the OE+NC group (149 ± 2.91) was higher than that in the NC+NC group (131 ± 4.61, p < 0.05). The number of migratory cells in the KD+NC group (29 ± 4.50) was lower than that in the NC+NC group (p < 0.05), and the number of migratory cells in the KD+OE group (89 ± 3.04) was partially restored as compared with that in the KD+NC group (p < 0.05, Figure 5D, 5E). The Transwell assay showed that the migration function of HCC cells was inhibited by *CCNB2* silencing, and the overexpression of *JAG1* partially restored the inhibition of migration ability following *CCNB2* silencing. Thus, the Transwell assay further confirmed that *CCNB2* promotes cell migration by increasing *JAG1* expression.

### *JAG1* expression is up-regulated in HCC tissues

**Figure 6:**
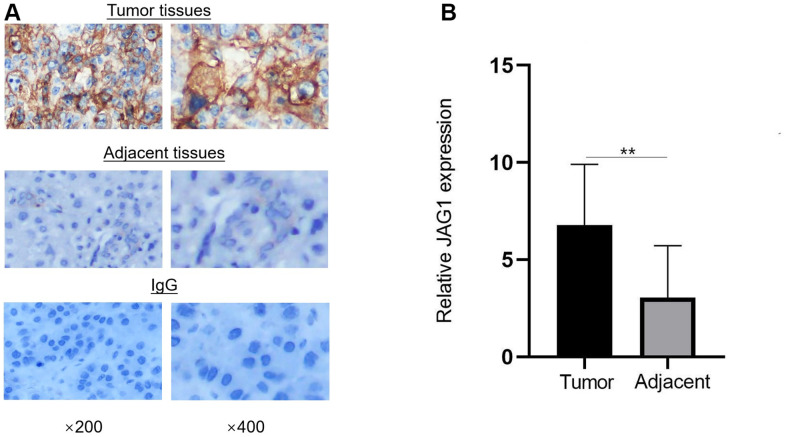
**JAG1 is overexpressed in HCC tissues.** (**A**) Immunohistochemistry showed the expression of JAG1 in HCC and adjacent tissues. (**B**) The expression level of JAG1 was significantly higher in HCC tissues than in adjacent tissues. **p < 0.01.

IHC revealed that among the 82 patients with HCC, 56 patients (68.29%) showed high expression of JAG1, while 26 patients (31.71%) showed low expression of *JAG1*. *JAG1* expression was low in 17 patients (85%) and high in three patients (15%) with paracancerous lesions (Figure 6A). The expression of JAG1 was higher in HCC tissues (6.78 ± 3.12) than in the adjacent tissues (3.05 ± 2.67), and the difference was statistically significant (p = 0.000, Figure 6B). Studies on the correlation between the expression of *JAG1* and clinicopathological indicators showed that the expression of *JAG1* was significantly correlated with tumour size and number, and tumour thrombus formation, but not associated with gender, age, hepatitis B virus infection, Child-Pugh class, alpha fetoprotein level and tumour stage and grade (Table 2).

**Table 2:** Relationship between JAG1 and clinicopathological features of HCC patients(n=82).

**Parameters**	**JAG1**		***Х^2^***	***P***
	**Low**	**High**		
Sex			2.299	0.257
Male	26	48		
Female	5	3		
Age (year)			0.138	0.710
<60	16	34		
≥60	9	23		
HBsAg			0.033	0.855
Positive	24	43		
Negative	5	10		
Child-pugh			0.553	0.740
A	23	40		
B	5	9		
C	1	4		
Cirrhosis			1.931	0.165
Positive	18	26		
Negative	10	28		
AFP (ng/mL)			2.079	0.149
<400	22	42		
≥400	3	15		
Tumor size (cm)			21.011	0.000*
<5	21	15		
≥5	5	41		
Tumor number			14.112	0.000*
=1	11	45		
≥2	16	10		
Tumor thrombus			17.912	0.000*
Positive	14	6		
Negative	12	50		
TNM stage			0.757	0.624
I- II	22	51		
III-IV	4	5		
Differentiation			2.726	0.210
Low to medium	35	40		
High	1	6		

*p<0.05 was considered statistically significant.

## DISCUSSION

An in-depth study of the mechanism of HCC development is crucial for its efficient diagnosis and treatment [9]. The cell cycle and its regulation play an essential role in a cell’s life, and this has been a hot topic of research on tumours for the past few years [15]. Various factors that cause abnormalities in the cyclin-CDK complex may imbalance the cell cycle regulation and further induce cell canceration [15, 16]. Previous studies have reported that *CCNB2* is overexpressed in bladder carcinoma [17], breast carcinoma [18] and lung adenocarcinoma [19]. The detection of circulating *CCNB2* mRNA in serum may be clinically relevant for monitoring tumour metastasis and for screening treatment methods [20]. Bioinformatics analysis has shown that *CCNB2* is up-regulated in HCC and is significantly related to the survival and prognosis of patients [21]. Our previous study showed that *CCNB2* is overexpressed in HCC tissues and is correlated with patient prognosis. Silencing the *CCNB2* gene inhibited the proliferation, migration and G2/M phase progression of HCC cells and induced their apoptosis [8].

To further study the function of *CCNB2* in HCC, we conducted a tumour formation experiment in nude mice. We found that knockdown of the *CCNB2* gene inhibited tumorigenesis, while inhibition of *CCNB2* knockdown increased tumour volume and weight; this finding suggested that *CCNB2* may promote the occurrence and development of HCC. *In vivo* experiments further confirmed that *CCNB2* is an oncogene that promotes the proliferation of HCC cells.

Proteomics can reveal the related pathways of disease occurrence and development and enable to explore new drug targets, biomarkers and signalling pathways [22]. In the present study, proteomic techniques were used to investigate the potential role of CCNB2 in HCC cell lines. A total of 130 differentially expressed proteins were found, and 30 proteins were selected for PRM analysis. The following significant differentially expressed proteins were screened: RAB5A, PPCS and JAG1. IPA results showed that JAG1 was associated with the proliferation and apoptosis of HCC cells. JAG1 could probably be the target of CCNB2, and it was selected for further experimental confirmation.

JAG1 is a cell surface ligand that functions in the Notch signalling pathway, which is crucial in cellular activity [23]. *JAG1* overexpression has been detected in various cancers and is related to poor clinical prognosis [24]. A novel class of Notch inhibitors that block *JAG1* has been shown to limit tumour angiogenesis [25]. *JAG1* is significantly up-regulated in gastric cancer [26] and breast cancer [27]. Previous studies have shown that *JAG1* is overexpressed in HCC cell lines and is associated with the shorter survival period of patients with HCC [28].

We used tumour tissues from nude mice for the WB assay and found that *CCNB2* was correlated with *JAG1* expression. CCK-8 and Transwell assays were performed to confirm the influence of *CCNB2* on the proliferation and migration of HCC cells. The results showed that silencing the *CCNB2* gene reduced the proliferation and migration of HCC cells, while *JAG1* overexpression partially restored the reduced proliferation and migration of HCC cells caused by *CCNB2* silencing. These results confirmed the significant functional correlation between *CCNB2* and *JAG1* and indicated that *CCNB2* may accelerate the occurrence and progression of HCC by activating *JAG1*.

*CCNB2* may regulate *JAG1* at the transcriptome or proteome level. Transcription and post-transcriptional regulation are two main regulation modes of gene expression, and their abnormal regulation is the cause of human diseases [29]. Transcriptional regulation can affect lymph node metastasis, which is an important mechanism in the spread of cancer [30]. Regulation of protein mainly includes allosteric regulation, covalent modification, proteolytic cleavage and association of other regulatory proteins [31]. Abnormal protein phosphating regulation in covalent modification will lead to various diseases, including tumors [32].

Finally, IHC showed that JAG1 was overexpressed in HCC tissues and was correlated with tumour size and number and tumour thrombus formation. The liver is rich in blood vessels, and JAG1 overexpression may regulate the metastasis and prognosis of HCC. Metastasis leads to poor efficacy and recurrence of HCC in patients.

In conclusion, the present study confirmed the effect of *CCNB2* on tumorigenesis of HCC and its possible mechanism. *CCNB2* may regulate the expression of *JAG1* to play a role in HCC proliferation, thus providing a new insight for further studies on the pathogenesis of HCC.
